# The evolution of brain neuron numbers in amniotes

**DOI:** 10.1073/pnas.2121624119

**Published:** 2022-03-07

**Authors:** Kristina Kverková, Lucie Marhounová, Alexandra Polonyiová, Martin Kocourek, Yicheng Zhang, Seweryn Olkowicz, Barbora Straková, Zuzana Pavelková, Roman Vodička, Daniel Frynta, Pavel Němec

**Affiliations:** ^a^Department of Zoology, Faculty of Science, Charles University, CZ-12844 Prague, Czech Republic;; ^b^Prague Zoo, CZ-17100 Prague, Czech Republic

**Keywords:** intelligence, cognition, evolution, brain size, number of neurons

## Abstract

Reconstructing the evolution of brain information-processing capacity is paramount for understanding the rise of complex cognition. Comparative studies of brain evolution typically use brain size as a proxy. However, to get a less biased picture of the evolutionary paths leading to high cognitive power, we need to compare brains not by mass but by numbers of neurons, which are their basic computational units. This study reconstructs the evolution of brains across amniotes by directly analyzing neuron numbers by using the largest dataset of its kind and including essential data on reptiles. We show that reptiles have not only small brains relative to body size but also low neuronal densities, resulting in average neuron numbers over 20 times lower than those in birds and mammals of similar body size. Amniote brain evolution is characterized by the following four major shifts in neuron–brain scaling. The most dramatic increases in brain neurons occurred independently with the appearance of birds and mammals, resulting in convergent neuron scaling in the two endotherm lineages. The other two major increases in the number of neurons happened in core land birds and anthropoid primates, which are two groups known for their cognitive prowess. Interestingly, relative brain size is associated with relative neuronal cell density in reptiles, birds, and primates but not in other mammals. This has important implications for studies using relative brain size as a proxy when looking for evolutionary drivers of animal cognition.

## Significance

The evolution of brain processing capacity has traditionally been inferred from data on brain size. However, similarly sized brains of distantly related species can differ in the number and distribution of neurons, their basic computational units. Therefore, a finer-grained approach is needed to reveal the evolutionary paths to increased cognitive capacity. Using a new, comprehensive dataset, we analyzed brain cellular composition across amniotes. Compared to reptiles, mammals and birds have dramatically increased neuron numbers in the telencephalon and cerebellum, which are brain parts associated with higher cognition. Astoundingly, a phylogenetic analysis suggests that as few as four major changes in neuron–brain scaling in over 300 million years of evolution pave the way to intelligence in endothermic land vertebrates.

The evolution of cognitive capacity or “intelligence” and its underlying neural substrate has been of long-standing interest to biologists. Great strides have been made in understanding the evolution of brain size in vertebrates, with studies analyzing data on thousands of species (1–3). Since larger animals have larger brains but are not necessarily smarter, most studies of cognitive evolution use relative brain size (corrected for body size), which is thought to reflect extra neurons beyond those needed for controlling the body (4). We now have a good idea where major changes in brain–body scaling happened within birds (2) and mammals (3), and it is also clear that both mammals and birds have relatively larger brains than nonavian sauropsids (hereafter referred to as reptiles), although this has been rarely formally quantified because data on reptilian brain sizes are scarce (5).

However, we still lack a clear picture of the evolution of actual brain processing capacity. This is because the same increase in relative brain size can be reached by different evolutionary paths, not always involving actual brain enlargement, and might often result from selection on body size (3). Moreover, similarly sized brains of distantly related species can harbor substantially different numbers of neurons overall and in major brain parts (6, 7). These two caveats invalidate the very idea that we can estimate extra neurons and glean information about cognitive capacity from absolute or relative brain size alone.

This capacity is better determined by the number of neurons in the brain or specific brain parts (although their relative importance is still debated), their connections, interneuronal distance, and axonal conduction velocity (8, 9). Unlike brain size, though, these measures are not readily available for a sufficient number of species to be of practical use. Nevertheless, thanks to methodological advances (10), neuronal scaling rules (the allometric relationship between brain mass and neuron numbers) have now been determined for eight high-level mammalian clades (6, 11–13) as well as for a limited sampling of birds (14, 15).

To get the big picture of amniote brain evolution, we have to include data on nonavian reptiles. The deepest split in amniote evolution occurred between the synapsid lineage, leading to mammals, and the sauropsid lineage, including reptiles and birds. We cannot tell if similarities between birds and mammals are due to shared ancestry or convergent evolution without considering reptiles. Yet, the dearth of quantitative data on reptile brains is striking—brain mass is available for 183 species (5, 16), compared to thousands for birds and mammals, and neuron numbers are known for a mere 4 reptile species (17–19).

Taken together, to understand the evolution of brain processing capacity in amniotes, we need to include nonavian reptiles, consider changes in both brain–body and neuron–brain scaling, and examine the allocation of neurons to different brain parts. In this study, we provide these much needed data and reconstruct the big picture of brain evolution in amniotes in terms of neuron numbers.

## Results

**Fig. 1.**
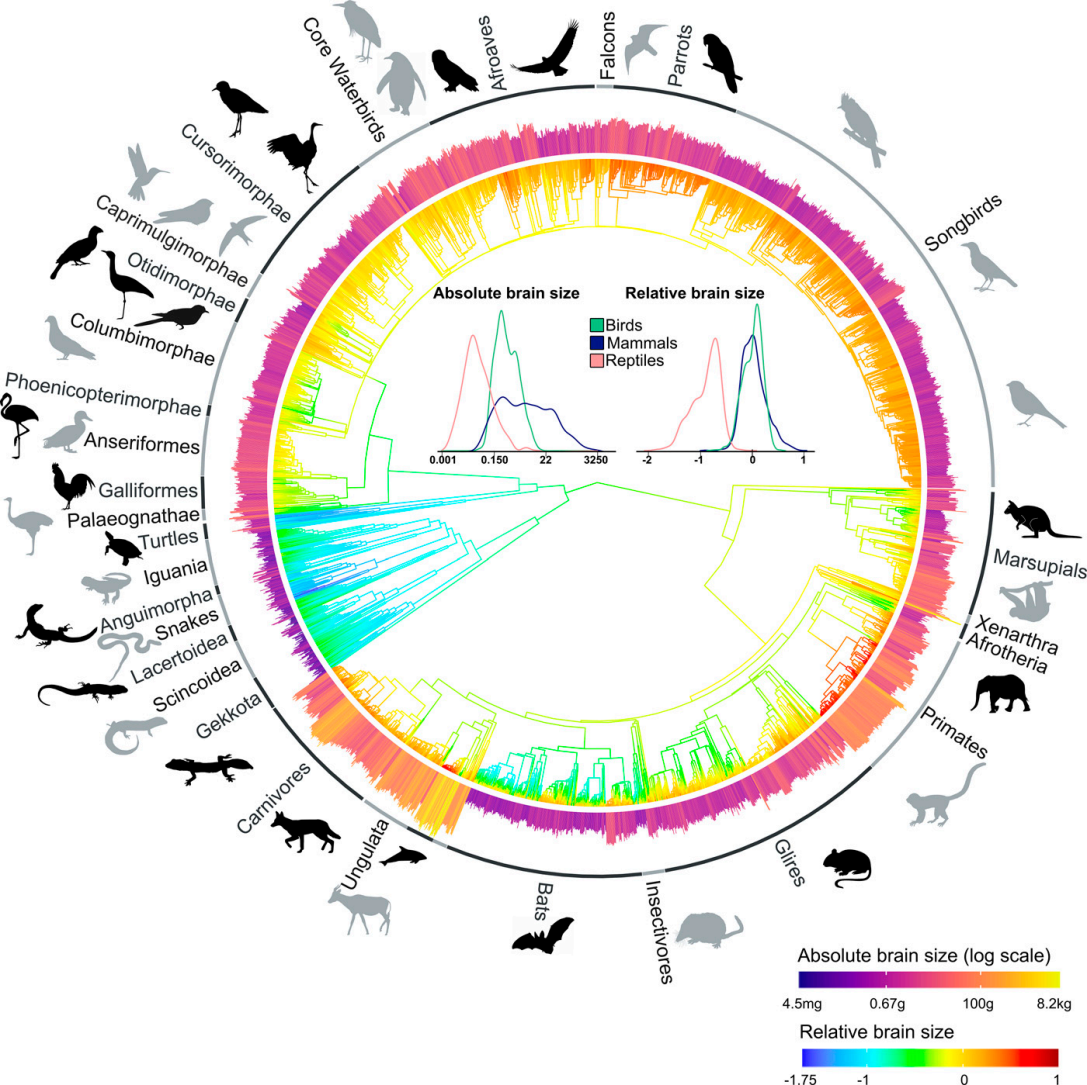
Absolute and relative brain sizes mapped to amniote phylogeny. Relative brain size expressed as residuals from PGLS regression of log-transformed brain mass on log-transformed body mass is mapped on the tree, with the internal nodes showing relative brain sizes based on an ancestral reconstruction of brain and body mass. The outer bars represent log-transformed absolute brain mass. The *Inset* graphs show the density distribution of absolute and relative brain sizes in birds, mammals, and nonavian reptiles. Silhouette illustrations are from phylopic.org (see *SI Appendix* for detailed credits).

**Fig. 2.**
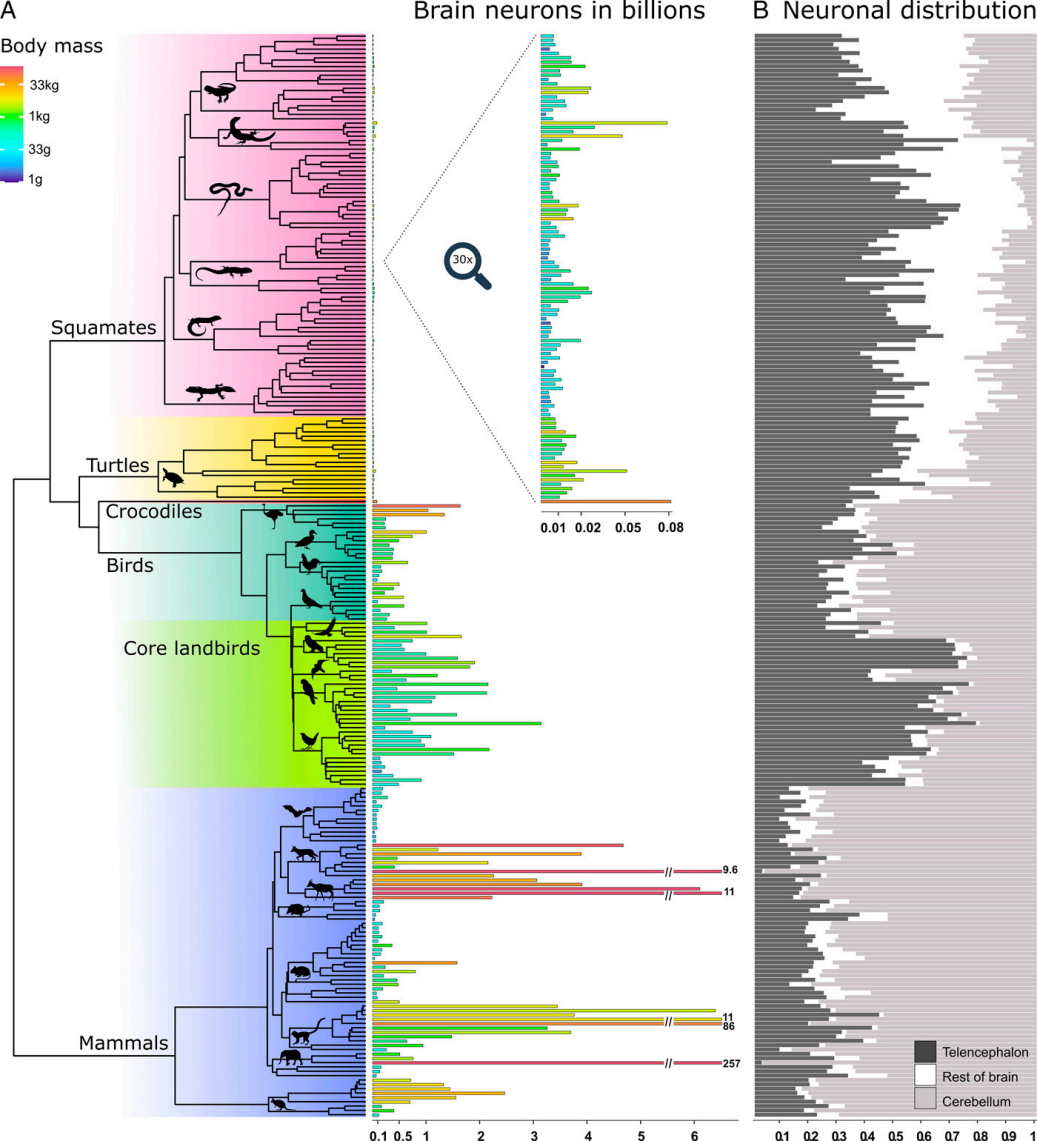
Absolute brain neuron numbers in amniotes and their allocations to major brain parts. (*A*) Absolute brain neuron numbers are plotted on the amniote phylogenetic tree. Bar lengths correspond to neuron numbers (note that the bars for the five species with the highest numbers of neurons have been truncated), while bar color indicates body mass on a logarithmic scale. The bars for reptiles have been enlarged 30 times in the *Inset*. (*B*) Allocation of total brain neurons to major brain parts. The gray-scale bars indicate the proportion of brain neurons found in the telencephalon, rest of brain, and cerebellum. Cerebellum is the dominant fraction in all mammals, while there are two distinct patterns in birds, with cerebellum being predominant in basally diverging birds and telencephalon in core land birds (Telluraves). In reptiles, the cerebellum generally contains fewer neurons than the rest of brain, which accounts for a negligible fraction of brain neurons in endotherms. An evolutionary trend of increasing cerebellar neuronal fraction is seen in turtles and crocodiles. Silhouette illustrations are from phylopic.org (see *SI Appendix* for detailed credits).

Using the isotropic fractionator (10), we quantified brain cellular composition in 107 species of squamate reptiles and turtles, covering all major lineages and a wide range of body sizes, and in an additional 37 species of birds. We then combined this with previously published data on birds, mammals, and reptiles, resulting in the largest dataset of vertebrate neuron numbers to date, comprising 251 species. Additionally, we compiled data on brain and body sizes in almost 4,000 species of amniotes, including 312 species of reptiles. Mapping these quantitative traits on a time-calibrated phylogenetic tree reveals that birds and mammals have convergently increased both absolute and relative brain size (Fig. 1) and neuron numbers (Fig. 2*A*), resulting in a disproportionate expansion of brain processing capacity. While there is substantial overlap among the distributions of absolute brain sizes in all three groups, relative brain sizes are almost entirely distinct in reptiles (Fig. 1), with birds and mammals having on average about sixfold and eightfold larger brains, respectively, than expected for a reptile with the same body mass (*SI Appendix*, Table S1). Importantly, this increase in brain size goes hand in hand with an increase in neuron density (number of neurons per brain structure mass), even though, across amniotes, neuron densities go down as brains get larger (*SI Appendix*, Fig. S1). The difference in nonneuronal (glial) cells is much less pronounced, although reptiles still show lower numbers (*SI Appendix*, Fig. S2*B*). As a result, reptiles have dramatically lower neuron numbers for a given body size. On average, birds and mammals harbor about 21- and 20-fold more neurons in their brains, respectively, than would be expected for equivalently sized reptiles (*SI Appendix*, Fig. S2 and Table S1). As an illustrative example, the squamate reptile with the most neurons in our dataset, the Asian water monitor, with a body mass of 3.9 kg, has 78 million brain neurons, which is comparable to the 168-g Golden hamster (84 million) or the 44-g King quail (80 million). The 90-kg Nile crocodile has only one-half as many neurons (83 million) as the 4.5-g goldcrest (164 million), to mention an extreme example.

These differences are not homogenous across brain regions. Not only can brains of the same size differ in the number of neurons but also the total number of neurons can be allocated to different brain parts. Here, we divided the brain into three parts, namely, the telencephalon, cerebellum, and the “rest of brain,” comprising the diencephalon, mesencephalon, and medulla oblongata. While the telencephalon has traditionally taken center stage as the “seat of higher cognition,” it is the cerebellum that accounts for most of this striking increase in neuron numbers. Birds and mammals have on average about 17- and 9-fold more neurons, respectively, in the telencephalon than expected for reptiles of equivalent body mass, but about 45- and 69-fold more neurons in the cerebellum. In the rest of brain, however, this amounts to about a ninefold and fourfold increase, which is less than the increase in relative brain size in mammals (*SI Appendix*, Table S1). Consequently, the allocation of brain neurons to the three major brain parts is distinct in reptiles, mammals, basally diverging birds, and core land birds (Fig. 2*B*). The ratio between telencephalic and cerebellar neurons varies among reptilian and avian groups but remains similar across mammals, implying the previously reported coordinated scaling of neurons in these structures (20) is specific to mammals.

**Fig. 3.**
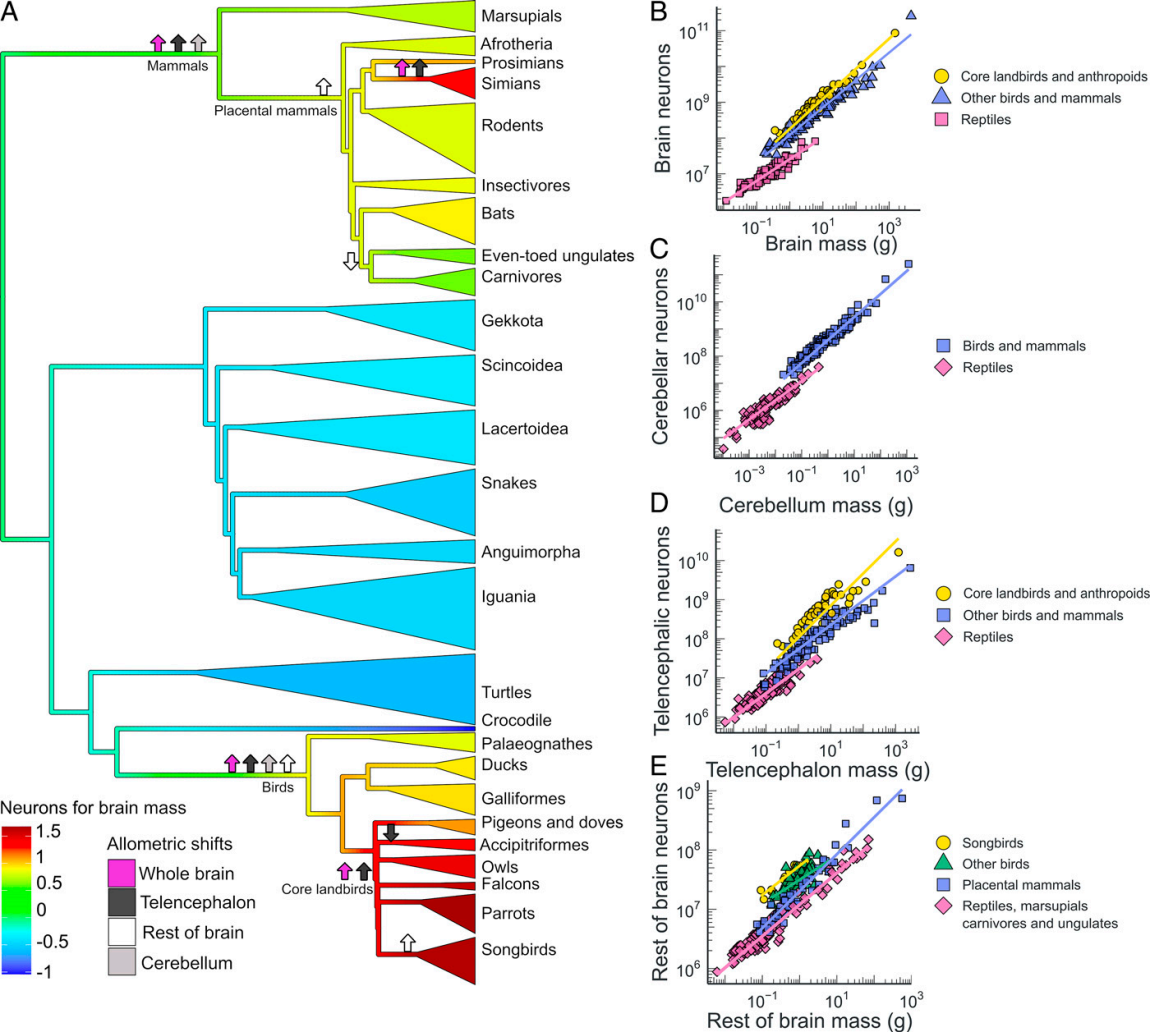
Shifts in neuron–brain scaling in amniotes and scaling of convergent allometric regimes. (*A*) Tree colors correspond to neuron density in the whole brain, with blue colors indicating low density and red colors high density. The arrows indicate the branches with shifts in allometric relationship between structure mass and neuron number (resulting in either an increase in neurons, arrow up; or a decrease in neurons, arrow down) for the whole brain, telencephalon, cerebellum, and rest of brain, identified by reversible-jump Markov chain Monte Carlo analysis with posterior probability of >0.7 for clades including more than three species. (*B*–*E*) Log-log plots of neuron number for structure mass with regression lines for the distinct regimes identified by PGLS analysis.

To further explore these changes in neuron scaling rules across amniotes, we fitted Bayesian reversible-jump bivariate multiregime Ornstein–Uhlenbeck models (21), which allow for the automatic detection of significant shifts in allometry (slope and intercept) on a phylogeny without the need to specify the shift locations a priori. These analyses identified several major macroevolutionary shifts in neuron scaling within amniotes (Fig. 3*A* and *SI Appendix*, Figs. S3–S6). Consistently, for the whole brain and major brain parts, the shifts were uncovered at the branches leading to mammals and birds, with the exception of the rest of brain, where the shift was located on the branch leading to placental mammals, assigning marsupials to the ancestral condition. Additional shifts happened in core land birds (comprising hawks and eagles, owls, falcons, songbirds, and parrots in our dataset) and anthropoid primates (monkeys and apes). The relatively low number of transitions to different optima in over 300 million years of evolution implies strong constraints are in place.

**Fig. 4.**
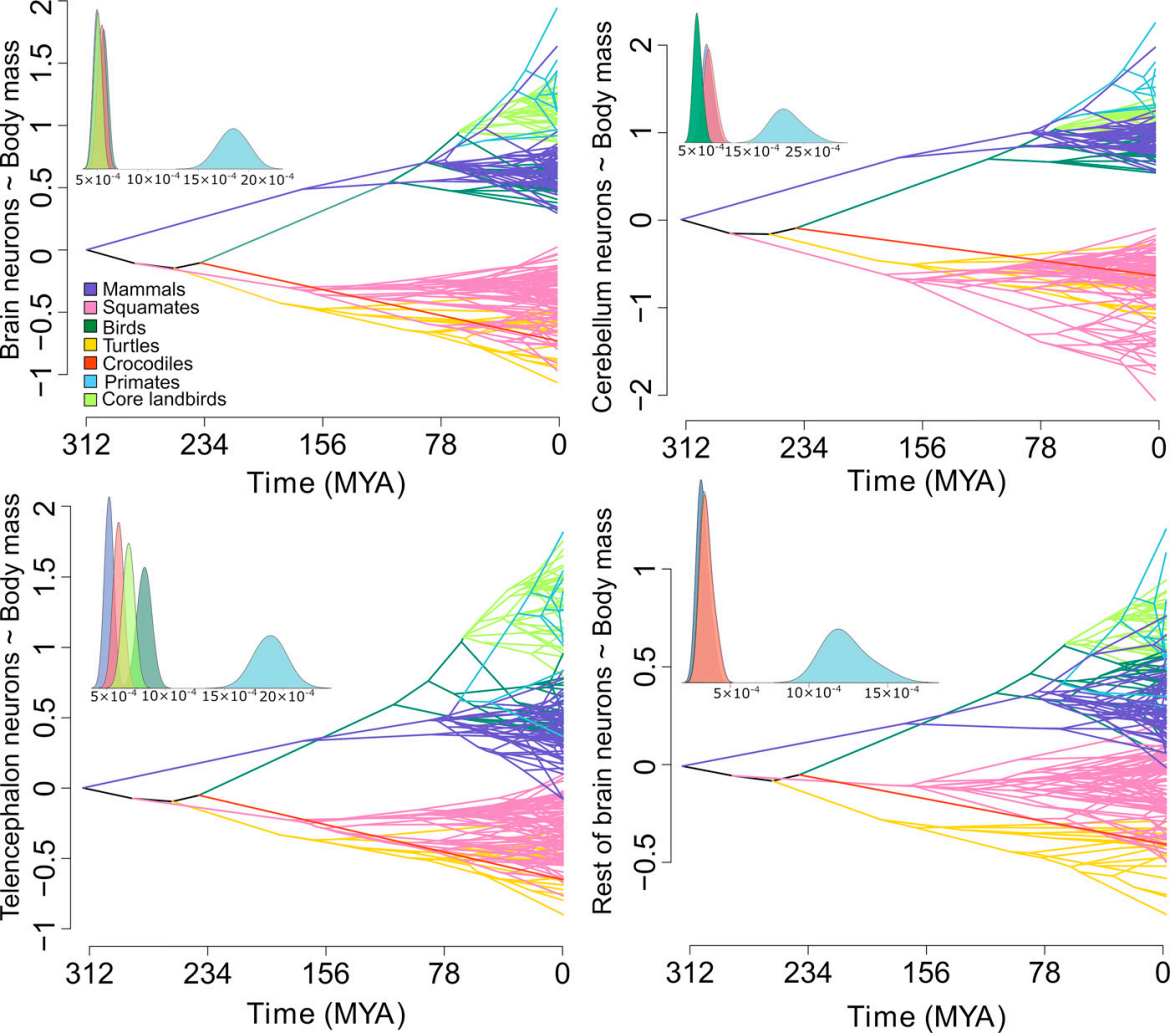
Phenograms showing the evolution of brain neuron numbers relative to body mass over time. Colors in phenograms correspond to major reptile lineages (squamates, turtles, crocodiles), primates and core land birds (the groups identified as having experienced significant shifts in allometric scaling), and other birds and mammals. The *Inset* graphs show the σ^2^ for residuals from PGLS as estimated by a multiple-variance Brownian motion model, corresponding to the strength of allometric integration. All nonavian reptiles are grouped in red color. Primates are characterized by weaker allometric integration of the number of neurons with body mass relative to all other groups.

To confirm these shifts and to determine whether they result in distinct or convergent allometric regimes, we tested the differences in the slope and intercept of the putative grades in a phylogenetic least squares (PGLS) framework (*SI Appendix*, Table S2). The best fit model for whole-brain neuron scaling comprises three groups, namely, reptiles, anthropoid primates and core land birds, and other birds and mammals, with similar convergences in the scaling of individual brain parts (Fig. 3 *B*–*E*). Although emphasis has previously been placed on the differences, here, we show a remarkably similar pattern of evolution of neuronal scaling in birds and mammals, despite their different brain organization. However, the sampling is still far from complete, so additional scaling shifts might be uncovered in the future. No major shifts in brain neuron scaling were identified within nonavian reptiles, despite their long evolutionary history. Similar changes were uncovered for the scaling of neurons with body mass (*SI Appendix*, Fig. S7 and Table S3), where an additional decrease in the number of cerebellar neurons was found in snakes. This is due partly to their elongated bodies skewing the brain–body relationship and partly due to the reduction of the cerebellum, which is common to limbless squamates and associated with the pattern of locomotion (22). The resulting changes in the number of neurons for body mass follow different paths in different brain parts (Fig. 4 and *SI Appendix*, Fig. S8).

The above findings about the differential distribution of neurons to major brain parts in mammals are slightly complicated by the fact that the telencephalon was dissected differently in the mammalian studies; the number of telencephalic neurons, therefore, excludes the striatum in mammals, which is included with the rest of brain instead. The number of telencephalic neurons also excludes the olfactory bulbs (OBs) in 26 species of mammals because they were not available for analysis. The results are unlikely to be significantly affected by this difference in brain division or the missing OBs, as the striatum accounts for a small fraction of telencephalic neurons and OBs account for a small proportion of brain neurons in these mammalian groups (in the 56 species with OB available, 0.02 to 15%; mean, 5%). Nevertheless, we repeated the analysis with data including estimates of OB mass and neuron number and estimates for the striatum added to the telencephalon and subtracted from the rest of brain. These corrections resulted in an average 5.5% increase in estimated telencephalic neurons and a 26% decrease in estimated rest of brain neurons in mammals. This only strengthens the conclusion that the rest of brain contains a minor fraction of brain neurons in mammals and does not change the distinct grades identified by PGLS (*SI Appendix*, Tables S4 and S5). To further demonstrate that different brain division in mammals does not significantly affect the results, we compared numbers of neurons in the avian pallium (comprising the hyperpallium, mesopallium, nidopallium, arcopallium, and hippocampus) with its homolog—the mammalian pallium (comprising the neocortex, hippocampus, olfactory cortices such as piriform and entorhinal cortex, and pallial amygdala). This comparison confirms the convergences in neuron–body mass scaling between anthropoid primates and core land birds and between other birds and nonprimate mammals (*SI Appendix*, Fig. S8).

To measure the evolutionary flexibility of the scaling rules, we assessed the Brownian motion rate of evolution (σ^2^) of residuals from PGLS regressions and compared them among the allometric grades and brain parts. The stronger the allometric integration, the lower the residual variation and hence the rate of evolution. In other words, a high rate of evolution means that the scaling is not very tight and species can easily deviate in either direction. The strength of allometric integration was generally similar in all the analyzed clades, suggesting quick shifts between the different optima. Primates, however, show accelerated rates of evolution (*SI Appendix*, Tables S6 and S7), which is indicative of relaxed constraints or strong selection. The allometric integration in the cerebellum is strongest in birds, possibly due to the constraints of active flight, requiring a high number of cerebellar neurons, but precluding substantial brain enlargement. The rates of evolution are the same for telencephalon and cerebellum in mammals, but the cerebellum has a 1.5-fold higher rate in reptiles, whereas in birds, the telencephalon has a 3-fold higher rate than the cerebellum (*SI Appendix*, Table S8).

**Fig. 5.**
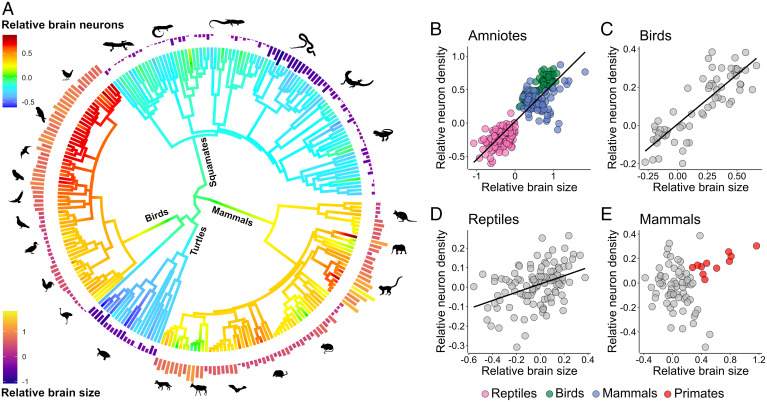
Large relative brain size tends to co-occur with high relative neuron density across amniotes. (*A*) Ancestral reconstruction of the relative number of brain neurons for brain mass is mapped on a phylogenetic tree. The outer bars represent relative brain size (calculated as residuals from PGLS regression of brain mass on body mass across the amniote dataset). (*B*–*E*) Plots of relative neuron density against relative brain size calculated across amniotes (*B*), birds (*C*), reptiles (*D*), and mammals (*E*). When analyzed across all amniotes, there is a significant positive association between larger relative brain size and higher relative neuron density (*B*) (PGLS;t_249_ = 5.85, *P* < 0.001, λ = 0.92). This pattern holds also within birds (*C*) (PGLS;t_63_ = 6.01, *P* < 0.001, λ = 0.52) and reptiles (*D*) (PGLS;t_108_ = 3.74, *P* < 0.001, λ = 0.37), but not within mammals (*E*) (PGLS;t_74_ = 1.15, *P* = 0.25, λ = 0.96). However, primates show a positive association between the analyzed traits (PGLS;t_9_ = 2.77, *P* = 0.02, λ = 0.3; *SI Appendix*, Fig. S9). Silhouette illustrations are from phylopic.org (see *SI Appendix* for detailed credits).

Another general pattern emerged, revealing a significant positive correlation between relative brain size and relative neuron density (Fig. 5). This holds not only across amniotes (Fig. 5*B*), as a result of the differences between reptiles and endotherms, but also within birds (Fig. 5*C*) and reptiles (Fig. 5*D*), when examined separately. Mammals in general do not follow this pattern (Fig. 5*E*) but primates do (*SI Appendix*, Fig. S9). While absolute neuron density on its own is not a meaningful proxy of brain processing capacity, as it predictably goes down with increasing brain size, relative neuron density (higher or lower than expected for a given brain size) is more informative. Just as animals with a large relative brain size will have larger brains for a similar body size, animals with higher relative neuron density will have more neurons in a similarly sized brain. These effects then compound in the taxa that exhibit a positive association between relative brain size and relative neuron density, leading to disproportionately higher numbers of neurons in species with relatively large brains. The same relative brain size can result from different processes, which do not necessarily involve selection for larger brains (3). Therefore, a simultaneous increase in relative brain size and neuron density might reflect selection on brain processing capacity (absolute number of neurons) and differentiate from passive changes due to body size. This association also suggests that if relative brain size is to be used, it might be a more appropriate proxy for cognitive capacity in birds than in mammals because, in birds, it might be an indirect measure of neuron numbers.

## Discussion

Phylogenetic analyses performed in this study have identified only four major shifts in neuron–body scaling in over 300 million years of amniote evolution. These occurred independently with the appearance of birds, mammals, core land birds, and primates. We suggest that these convergent increases in neuron numbers represent stepping stones in the evolution of avian and mammalian intelligence. No major shifts in the numbers of brain neurons were observed within nonavian reptiles, but they may have happened in other vertebrate groups. Relatively large brains have evolved several times in some cartilaginous and ray-finned fishes, while newts and salamanders have reduced brain size (23). It remains to be seen whether these changes in brain size are also accompanied by changes in neuronal density. At the moment, sufficient data on the numbers of neurons in amphibians and fishes are lacking; brain neurons have been quantified in two miniaturized species of ray-finned fish (24, 25) and two species of amphibians (26).

It is recognized that the energetic cost of neural tissue is an important constraint in the evolution of large brains (27). However, limited evidence exists for an association between relative brain size and metabolic rates within mammals or birds (28, 29). It has also been suggested that larger brains in endotherms compared to ectotherms can be attributed to their higher body temperatures and that increases in nonneuron cell numbers play a critical role (30). Here, we provide a different point of view. The massive increase in neuron numbers relative to body size in birds and mammals might have been enabled by actually relaxing the metabolic constraints due to the transition to endothermy. Since brain metabolism scales linearly with the number of neurons (31) and the brain carries a high energetic cost even at rest and in the absence of active signaling (32, 33), a high number of neurons constitutes an energy drain that cannot ever be truly turned off. Reptiles that rely heavily on energy conservation thus cannot afford this expensive tissue beyond a certain point, hence their low allometric exponent for the relationship between the number of neurons and body mass. With the adoption of endothermy, which is inherently metabolically expensive and requires a higher energy intake (34, 35), this constraint might have become relatively less important, enabling the rise in neuron numbers with a smaller percentage increase in total energy expenditure. The resulting increase in brain processing capacity, in turn, may have paid for itself in terms of improved foraging efficiency and other fitness benefits. Thus, the transition to endothermy might have tipped the balance of the cost/benefit ratio of neural tissue. Interestingly, one mammalian species in our dataset, the naked mole-rat, known for its low metabolic rate, weak ability to maintain stable body temperature, and high hypoxia tolerance (36, 37), also has a much lower than expected neuronal density in the telencephalon, which is comparable to those of reptiles.

The finding that cerebellar neurons account for most of the difference in neuron numbers between reptiles and endotherms is interesting in light of the mounting evidence that the cerebellum plays a key role in the evolution of sensorimotor and cognitive control of complex behaviors (38–41).

Despite their long independent evolution and distinct anatomical organization of the brain (42, 43), birds and mammals converged on similar numbers of telencephalic and cerebellar neurons, with yet another increase in telencephalic neurons seen in anthropoid primates and core land birds, which might provide the neural substrate for their remarkably similar cognitive feats (44). However, in contrast to mammals, where tight functional coupling and coordinated neuronal scaling of telencephalon and cerebellum are well established (20, 45), the evolution of a large, neuron-rich telencephalon in core land birds is not accompanied by a matching gain of cerebellar neurons. Given that the increase in neurons in the “smartest” birds is limited to the telencephalon, the cerebellum may turn out to be less important for cognitive functions in birds, although little evidence is available at the moment.

Interestingly, a higher relative brain size in birds is accompanied by a higher relative neuron density, whereas in mammals, no such relationship exists. This means that if we take two birds with equivalent absolute brain sizes, the one with the larger relative brain size will also likely have more neurons. This is in line with abundant evidence that relative brain size predicts intelligent behavior in birds (46–48). Ultimately, the cases where an increase in relative brain size is coupled to an increase in neuron density might indicate selection on the brain, as opposed to selection on body size. Primates are an exception among mammals in that they also seem to follow this pattern, suggesting the large brains of anthropoid primates are the result of selection on the neural substrate mediating their remarkable cognitive abilities. Moreover, primates show a weaker integration between neuron numbers and body size than other amniotes, a feature that likely contributed to the rapid evolution of their brains by increasing the variation that selection can act on (49).

This study highlights that encephalization trajectories, neuron densities, and neuron distribution to different brain parts can all be clade specific. Because of that, comparative studies of brain evolution should consider that changes in absolute and relative brain size might not translate directly into changes in brain processing capacity across different clades. A fruitful approach to the study of the evolution of cognition might be to combine the data on brain size, which are available for a broad range of living and fossil taxa, with data on neuron numbers and scaling, which give a more accurate picture of brain computing power.

## Methods

### Animals.

A total of 132 individuals of 107 species of reptiles and 91 individuals of 37 species of birds were used in this study. We aimed to cover the major lineages of squamate reptiles (Gekkota: 14 species, Scincoidea: 14 species, Lacertoidea: 15 species, Anguimorpha: 7 species, Iguania: 20 species, and Serpentes: 18 species) as well as the two major lineages of turtles (Pleurodira: 14 species, Cryptodira: 5 species) and include a wide range of body sizes. The bird species added in this study were from the following groups: Paleognathae (5 species) Galliformes (8 species), Anseriformes (7 species), Columbiformes (4 species), Accipitriformes (4 species), Strigiformes (6 species), and Falconiformes (3 species). Animals were preferentially wild caught, with those unavailable from the wild acquired from breeders and zoos. All animals were sexually mature or at least had adult-like size and coloration. The sex of all animals was determined upon dissection. Where possible, we preferentially collected animals of both sexes or males in the case of single individuals. However, based on previous findings, there are no significant sex differences in neuron numbers in either squamate reptiles (18) or birds (14), and intraspecific variation is negligible compared to the large scale of body and brain sizes in the sample.

### Ethical Approvals.

All procedures were approved by the Institutional Animal Care and Use Committee at Charles University (UKPRF/28830/2021), Ministry of the Environment of the Czech Republic (permission no. 53404/ENV/13-2299/630/13), Ministry of Culture of the Czech Republic (permission no. 47987/2013), and Ministry of the Environment of the Czech Republic (permission no. 53404/ENV/13-2299/630/13) and in compliance with the applicable legislation in the Czech Republic implementing the European guidelines (European Union directive no. 2010/63/EU) regarding the protection of animals used for scientific purposes.

### Perfusions.

The animals were killed by anesthetic overdose (intramuscular administration of ketamine and xylazine for reptiles; inhalation of halothane for birds except for ostrich, rhea, and emu, which were overdosed by intramuscular injection of anesthetics containing midazolam, detomidine, medetomidine, butorphanol, and ketamine). They were weighed and immediately perfused transcardially with warmed phosphate-buffered saline containing 0.1% heparin followed by cold phosphate-buffered 4% paraformaldehyde solution. Skulls were partially opened and postfixed for 30 to 60 min, after which brains were dissected and weighed. Brains were postfixed for additional 7 to 21 d and then dissected into parts and either processed immediately or transferred into antifreeze (30% glycerol, 30% ethylene glycol, 40% phosphate buffer) and kept frozen at −20 °C until processing.

### Brain Dissections.

Brains were dissected into major parts using the Olympus SZX 16 stereomicroscope. The cerebral hemispheres were detached from the diencephalon by a straight cut separating the subpallium from the thalamus. The cerebellum was cut off at the surface of the brainstem. The rest of brain refers to the remainder after separating the telencephalon and cerebellum, i.e., the diencephalon, mesencephalon, and medulla oblongata. For most individuals, only one cerebral hemisphere was processed since in our previous studies we detected negligible differences between left and right hemisphere mass and cell numbers. In birds, for one individual per species, the second hemisphere was dissected into pallium and subpallium. The hemisphere was embedded in agarose and sectioned on a vibratome at 300 to 500 μm (depending on the size of the hemisphere) in the coronal plane. Under oblique transmitted light at the stereomicroscope and with the use of a microsurgical knife (Stab Knife Straight; 5.5 mm; REF

7516; Surgical Specialties Corporation), we manually dissected the pallium from the subpallium on each section by cutting along the pallial–subpallial lamina, as defined by Reiner et al. (50). All the dissected parts were weighted to the nearest 0.1 mg using a Kern ALJ 120‐4 balance (Kern & Sohn GmbH).

### Isotropic Fractionator.

We estimated the total numbers of cells, neurons, and nonneuronal cells following the procedure of isotropic fractionator, as described earlier (10). Briefly, each dissected brain division was homogenized in 40 mmol sodium citrate with 1% Triton X-100 using Tenbroeck tissue grinders (Wheaton). When turned into an isotropic suspension of isolated cell nuclei, homogenates were stained with the fluorescent DNA marker 4′,6-diamidino-2-phenylindole dihydrochloride (DAPI) (Sigma-Aldrich), adjusted to a defined volume, and kept homogenous by agitation. The total number of nuclei in suspension, and therefore the total number of cells in original tissue, was estimated by determining the number of nuclei in 10-µL samples drawn from the homogenate. At least four aliquots were sampled and counted using a Neubauer improved counting chamber (BDH) at the Olympus BX51 equipped with epifluorescence and appropriate filter settings; additional aliquots were assessed when needed to reach the coefficient of variation among counts of ≤0.1. Once the total cell number was known, the proportion of neurons was determined by immunocytochemical detection of the neuronal nuclear marker NeuN (51). This neuron-specific protein was detected by a mouse monoclonal anti-NeuN antibody (clone A60, Chemicon, Temecula; dilution, 1:800) in birds and by a rabbit polyclonal anti-NeuN antibody (Merck Millipore; dilution, 1:800) in nonavian reptiles; the binding sites of the primary antibody were revealed by Alexa Fluor 546-conjugated goat anti-mouse immunoglobulin G (IgG) (Life Technologies, Carlsbad, CA; dilution 1:500) or Alexa Fluor 594-conjugated goat anti-rabbit IgG (Life Technologies, Carlsbad, CA; dilution 1:400), as appropriate. An electronic hematologic counter (Alchem Grupa) was used to count simultaneously DAPI-labeled and NeuN-immunopositive nuclei in the Neubauer chamber. A minimum of 500 nuclei was counted to estimate the percentage of double-labeled neuronal nuclei. Numbers of nonneuronal cells were derived by subtraction. Neuron density was calculated as the number of neurons in a given brain part divided by the brain part mass.

### Compilation of Data on Neuron Numbers.

In addition to data obtained in this study (Dataset S1), we included additional published data on neuron numbers in the Nile crocodile (17) and 2 species of anoles (19), 28 species of birds (14, 15), and 76 species of mammals (11–13, 52–60). The number of brain neurons and telencephalic neurons includes the OBs, except in 26 species of mammals, where they were not available (*Ursus arctos*, *Canis lupus familiaris*, *Mungos mungo*, *Hyaena hyaena*, *Felis cattus*, *Panthera leo*, *Cynomys* sp., *Macaca fascicularis*, *Macaca radiata*, *Papio cynocephalus*, *Homo sapiens*, *Sapajus apella*, *Saimiri sciureus*, *Amblysomus hottentotus*, *Dendrohyrax dorsalis*, *Dendrolagus goodfellowi*, *Macropus rufogriseus*, *Macropus parma*, *Macropus fuliginosus*, *Wallabia bicolor*, *Chaerephon pumilus*, *Coelura afra*, *Cardioderma cor*, *Hipposideros commersoni*, *Triaenops persicus*, *Miniopterus schreibersii*).

### Imputing OB and Striatum Values for Mammals.

For the analysis with corrections for missing OB and striatum included in the telencephalon, data were imputed in the following way: data on OB volumes and neurons were estimated using the appropriate scaling rules for the given clade (using data from 61 for volumes in carnivores, where OB were missing in most species). Data on striatum volume were available for 41 species in the dataset (12, 62–64), and for the remaining species, they were estimated from brain volume based on the average proportion in the respective group. Species-specific neuron densities were used to derive the number of striatal neurons, based on the fact that at least in mice (65) average cortical and striatal neuron densities are similar.

### Data on Brain and Body Mass.

We collected data on brain and body mass for 149 species of reptiles and supplied data on 3 additional species from the literature (66–68) (Dataset S2). We combined these with previously published datasets including 183 species of reptiles (5, 16), 1,989 species of birds (2), and 1,534 species of mammals (69). Endocranial volume was converted to brain mass by multiplying by the density of brain tissue (1.036 g/cm^3^) (70).

### Phylogeny.

For phylogenetic analyses, we adopted a phylogeny constructed from previously published species-level trees. We used recent published species-level time-calibrated phylogenies for squamates (71), birds (2), and mammals (72). For turtles and crocodiles, we used the Timetree of Life (73). We then stitched the trees together manually, using the divergence times from the Timetree of Life, and pruned them to match the brain size and neuron numbers datasets, substituting closely related species in a few cases that were not present in the published phylogenies.

### Data Analysis.

Analyses were performed in R version 4.0.3 (74) using average values for each species, and the variables were log10 transformed. Where appropriate, statistical significance was evaluated at an α level of 0.05.

#### Absolute and relative measures.

Absolute measures represent the species average value of the trait, while relative measures represent the residuals from PGLS regression across the group of interest (e.g., relative brain size in amniotes refers to the residuals from the PGLS regression of brain mass on body mass across amniotes with one slope and one intercept; relative neuron density in primates refers to the residuals from the regression of neuron number on brain mass across primates).

#### Ancestral reconstruction of brain and body sizes.

Ancestral reconstructions of continuous traits were performed using the function fastAnc in the package phytools v0.7 (75) and the function mvBM in the package evomap (76). Both methods gave very similar results, and only the values from fastAnc are used in the paper. The values were mapped onto phylogenetic trees using the R packages ggtree 2.4.0 (77) and phytools 0.7 (75).

#### Detection of significant shifts in allometric scaling.

We used the Bayesian reversible-jump bivariate multiregime Ornstein–Uhlenbeck modeling approach as implemented in the R package bayou 2.2.0 (21) to detect changes in the slope and intercept in neuron scaling in the whole brain and the three brain compartments with brain structure mass (effectively changes in relative neuron density) and body mass (reflecting changes in both neuron density and brain/structure size). This approach enables the identification of shifts in intercept and slope without specifying their location a priori.

We ran at least four chains with different random starting points for 10 million iterations, sampling every 100th iteration, and discarded the first 0.2 samples as burn-in. We used the following priors: half-Cauchy distribution with scale factor 0.1 for α (the strength of attraction toward an adaptive optimum) and σ^2^ (change of the trait per unit time), Poisson distribution with a mean equal to 2% of the total number of branches in the tree and a maximum number of shifts equal to 20% for the number of shifts, normal distribution θ∼N(µ = mean(trait), σ = 1.5 × SD(trait)) for the intercept, and normal distribution β∼N(µ = PGLS β, σ = 0.3) for the slope. We assessed the convergence of the run by inspecting the diagnostic plots and convergence of the chains using Gelman’s R-statistic (78) and by comparing the uncovered shift locations. We then combined the chains to summarize parameter estimates. All parameters had effective sample sizes greater than 150 (typically several thousand). Only shifts in clades containing more than three species were reported and included in further analysis.

#### PGLS analysis of allometric scaling.

We tested the models including the shifts identified in bayou in a PGLS framework, using the gls function in the R package nlme 3.1 (79), with Pagel’s lambda estimated using restricted maximum-likelihood. Separate slopes and intercepts were considered for the putative grades, i.e., the model was in the form Dependent∼Independent*Group. Model selection was carried out in a top-down fashion. Starting with the full model, the putative grades were consecutively merged with the ancestral grade to confirm they are significantly different, and the identified unrelated grades were then merged together to identify convergence. The models were compared using the core R function ANOVA, and the simplified model (with fewer factor levels) was adopted if the *P* value for the full model was >0.05. Shifts identified by bayou with a posterior probability of >0.7 were supported in all cases, except for the change in neuron density in the telencephalon of Accipitriformes.

#### Fold change estimates.

We used the following approach to quantify the fold change in the number of neurons for body size between reptiles and birds and mammals: as the regression lines are not parallel, differences in intercepts do not accurately capture the distance between the lines. We calculated the difference between the PGLS regression lines at every data point (body size of all included species) and then took the average. This corresponds to the average distance between the lines, i.e., the average fold change. See also *SI Appendix*, Fig. S10 for a visual representation.

#### Evolution rates.

To compare the evolutionary rates among the different brain parts (*SI Appendix*, Table S5), we used an approach devised specifically for comparing rates of evolution for multiple phenotypic traits on a phylogeny (80). The likelihood of a model with distinct evolutionary rates for each trait is compared to the likelihood of a model where all traits evolve at a common rate. We used the implementation in the function compare.multi.evol.rates in the R package geomorph 4.0.0 (81). The evolutionary rates of the groups with identified allometric shifts were compared using the function compare.evol.rates in geomorph 4.0.0. Here, we only tested primates as a whole, as they include anthropoid primates and the sample size is larger. The *P* values were calculated by bootstrap simulation with 10,000 iterations. The rates of evolution plotted in Fig. 3 are estimated using the multiple-variance Brownian motion framework (82) implemented in the mvBM function in the R package evomap (76) with Markov chain Monte Carlo sampling. Both methods agree in identifying primates as having significantly higher rates (weaker allometric integration) than the other groups.

## Supplementary Material

Supplementary File

Supplementary File

Supplementary File

## Data Availability

All study data are included in the supporting information and have also been deposited in Figshare, https://doi.org/10.6084/m9.figshare.
