# Supplementary material for: The evolution of brain neuron numbers in amniotes
Source: Proc Natl Acad Sci U S A. 2022 Mar 7;119(11):e2121624119. doi: 10.1073/pnas.2121624119 (PMC8931369; doi:10.1073/pnas.2121624119)
Supplement: Supplementary File [file pnas.2121624119.sapp.pdf]

PNAS
www.pnas.org

Supplementary Information for
The evolution of brain neuron numbers in amniotes

Kristina Kverková[1], Lucie Marhounová[1], Alexandra Polonyiová[1], Martin Kocourek[1], Yicheng Zhang[1], Seweryn Olkowicz[1], Barbora Straková[1], Zuzana Pavelková[1], Roman Vodička[2], Daniel Frynta[1], Pavel Němec[1*].

*Corresponding author: Pavel Němec
Email: pgnemec@natur.cuni.cz

This PDF file includes:

Figures S1 to S10
Tables S1 to S8

Other supplementary materials for this manuscript include the following:

Datasets S1 to S2

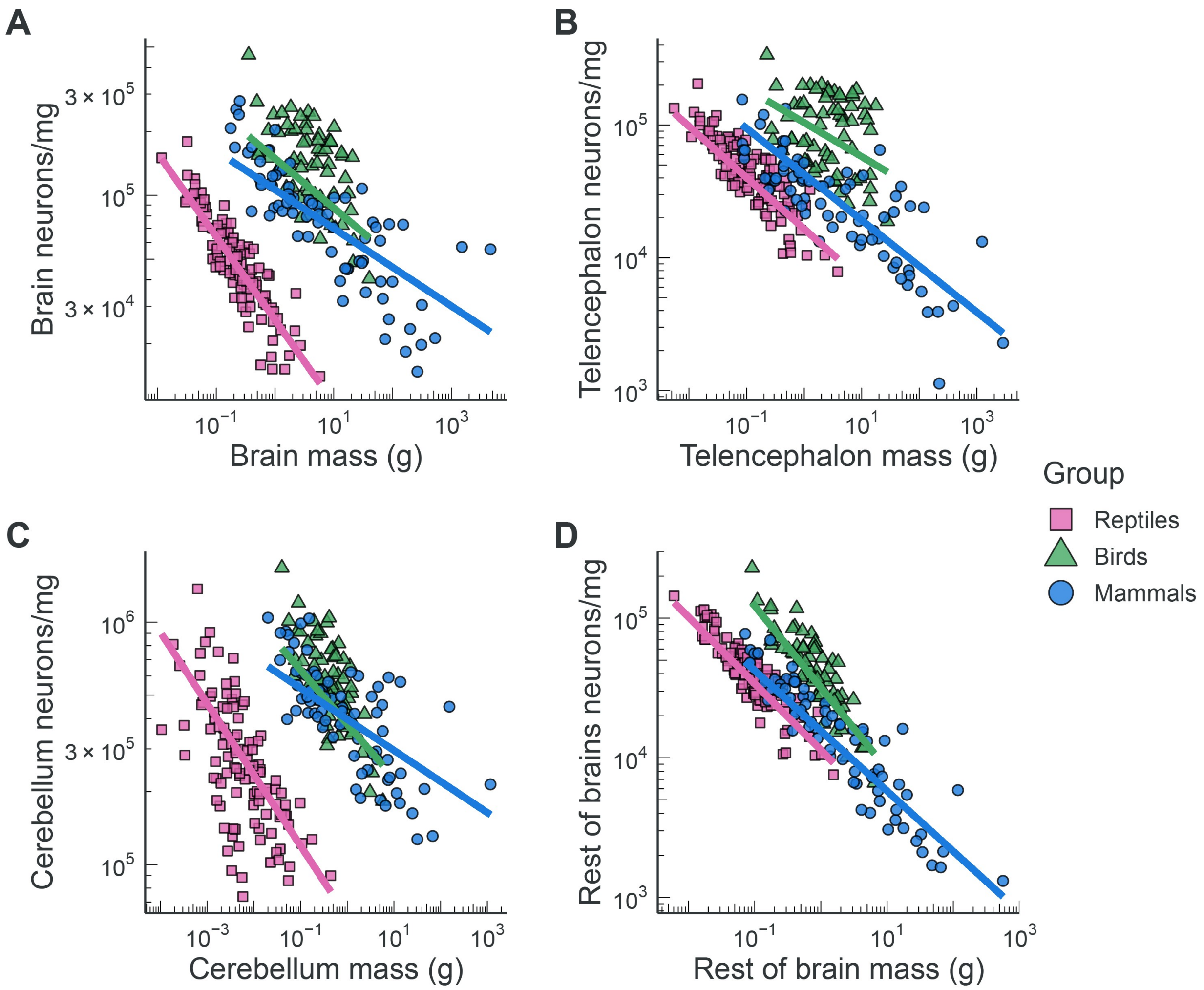

Supplementary Figure 1. Neuron densities go down with increasing brain structure mass across amniotes. Log-log plot of neuron densities against whole brain or brain part mass. The lines represent PGLS regression for the different groups.

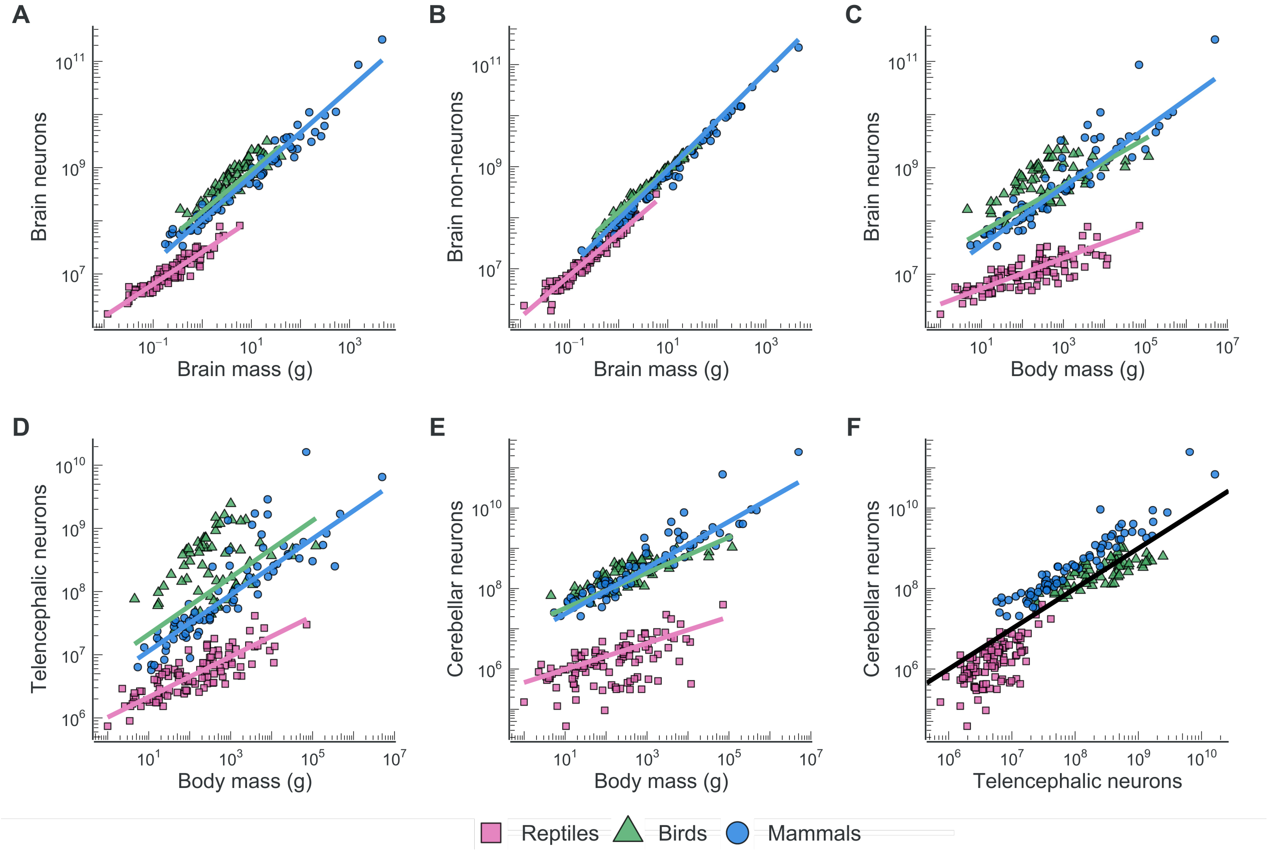

Supplementary Figure 2. Brain cellular scaling rules for birds, mammals and non-avian reptiles. (**A**, **B**) Scaling of the number of neurons with brain mass. The lines are PGLS regression lines. (**C-E**) Scaling of the number of neurons with body mass. The lines are PGLS regression lines. (**F**) Ratio of cerebellar to telencephalic neurons. The line represents a 1:1 ratio.

Brain neurons ~ Brain mass

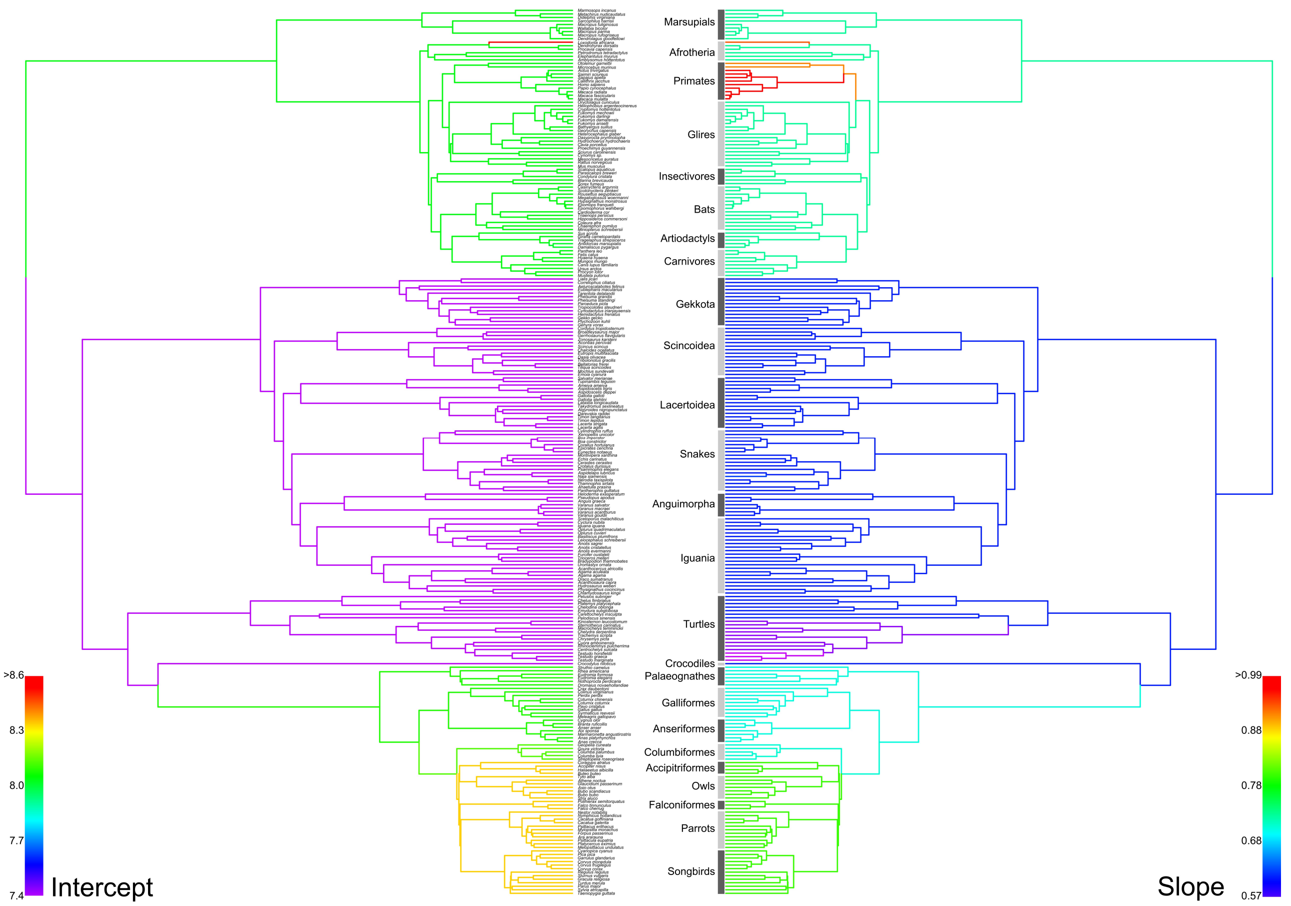

Supplementary Figure 3. Scaling of brain neurons with brain mass as estimated by bayou analysis for the 251 amniote species.

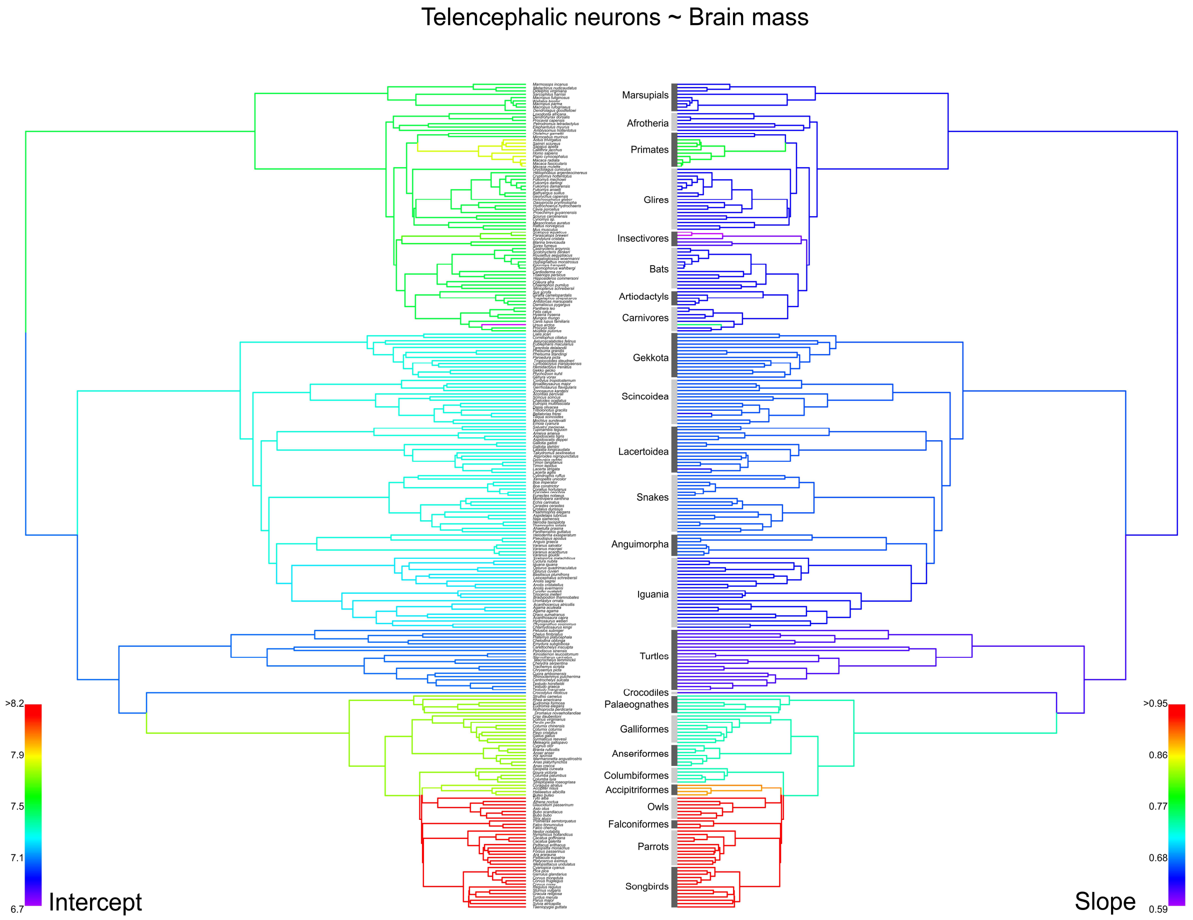

Supplementary Figure 4. Scaling of telencephalic neurons with brain mass as estimated by bayou analysis for the 251 amniote species.

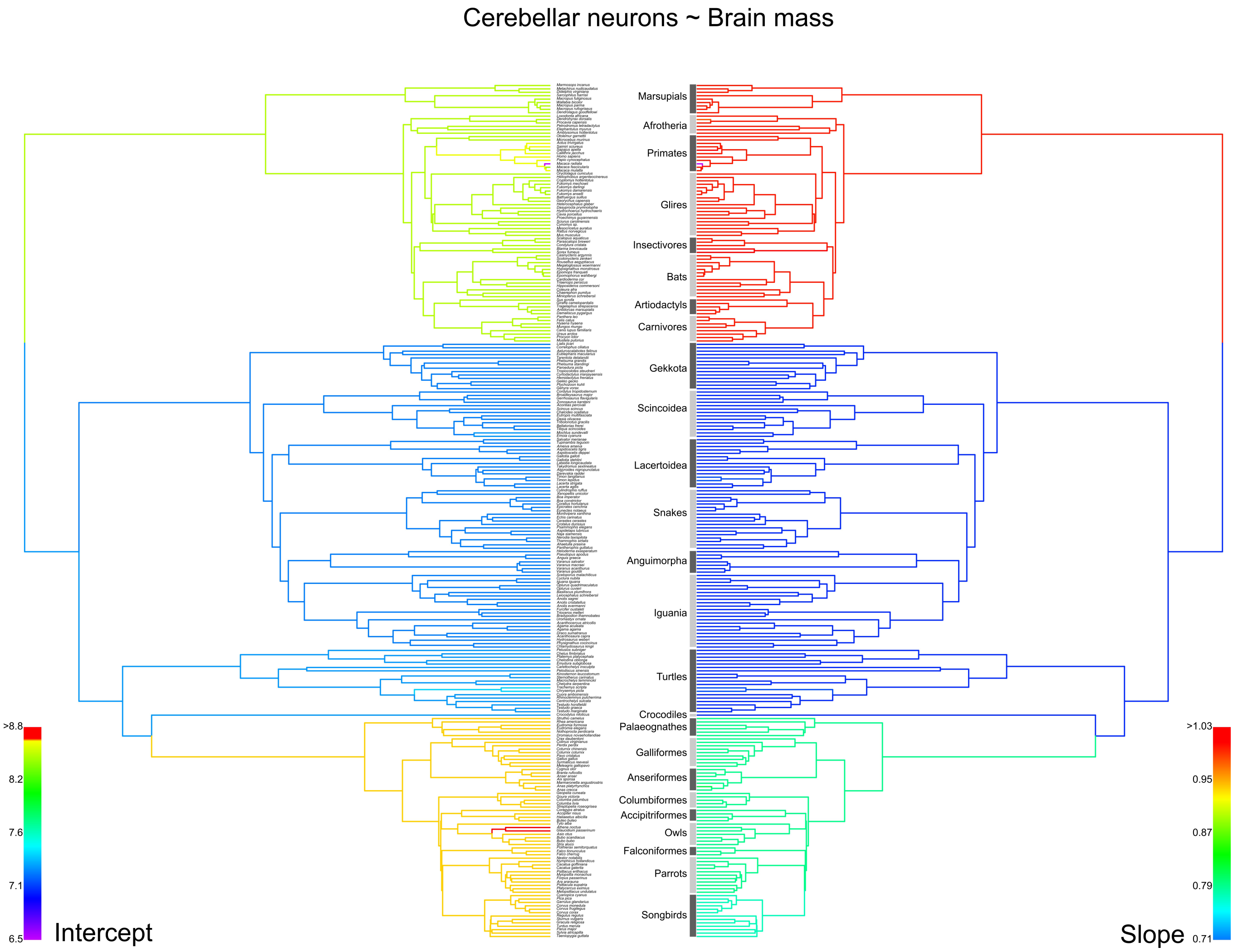

Supplementary Figure 5. Scaling of cerebellar neurons with brain mass as estimated by bayou analysis for the 251 amniote species.

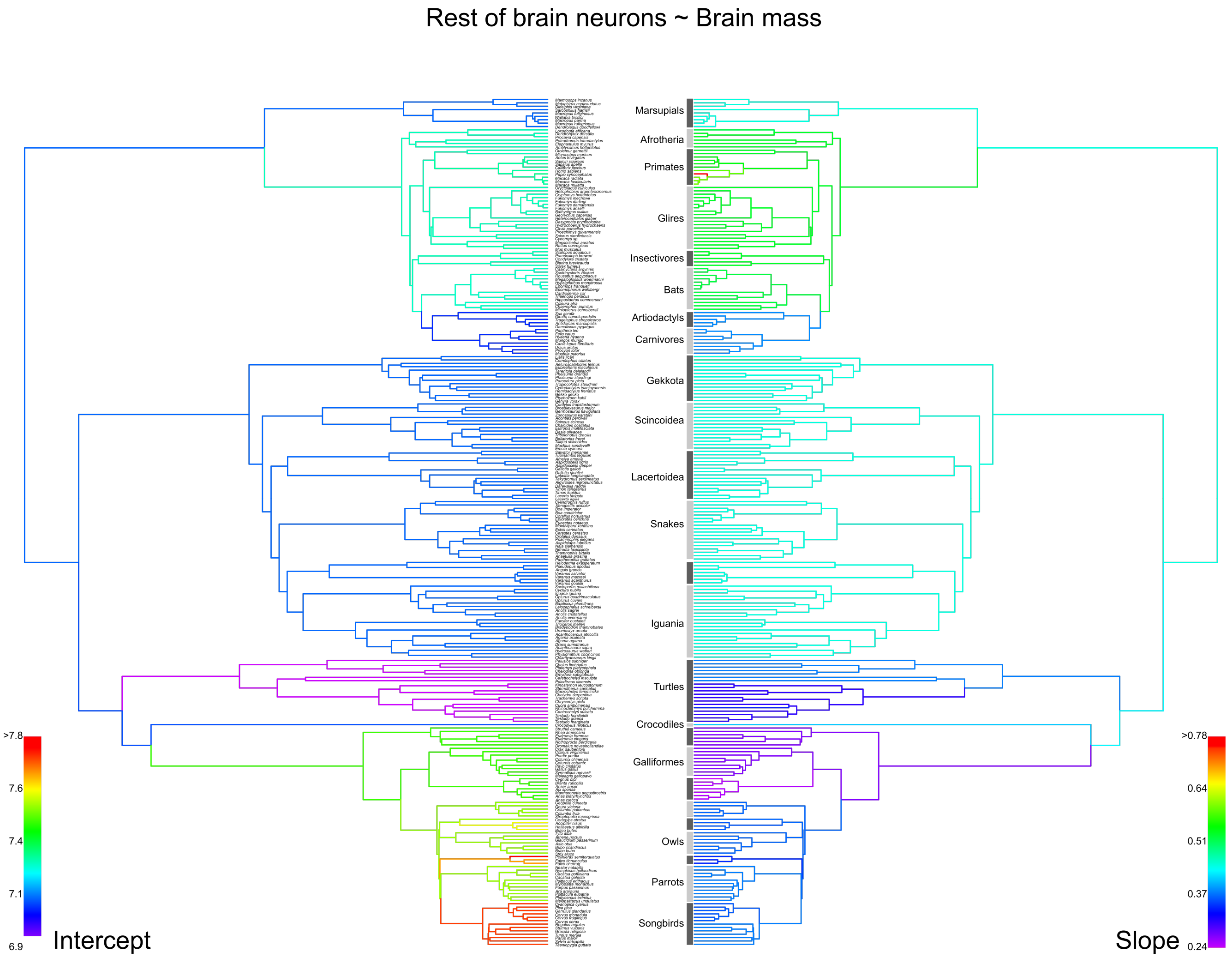

Supplementary Figure 6. Scaling of rest of brain neurons with brain mass as estimated by bayou analysis for the 251 amniote species.

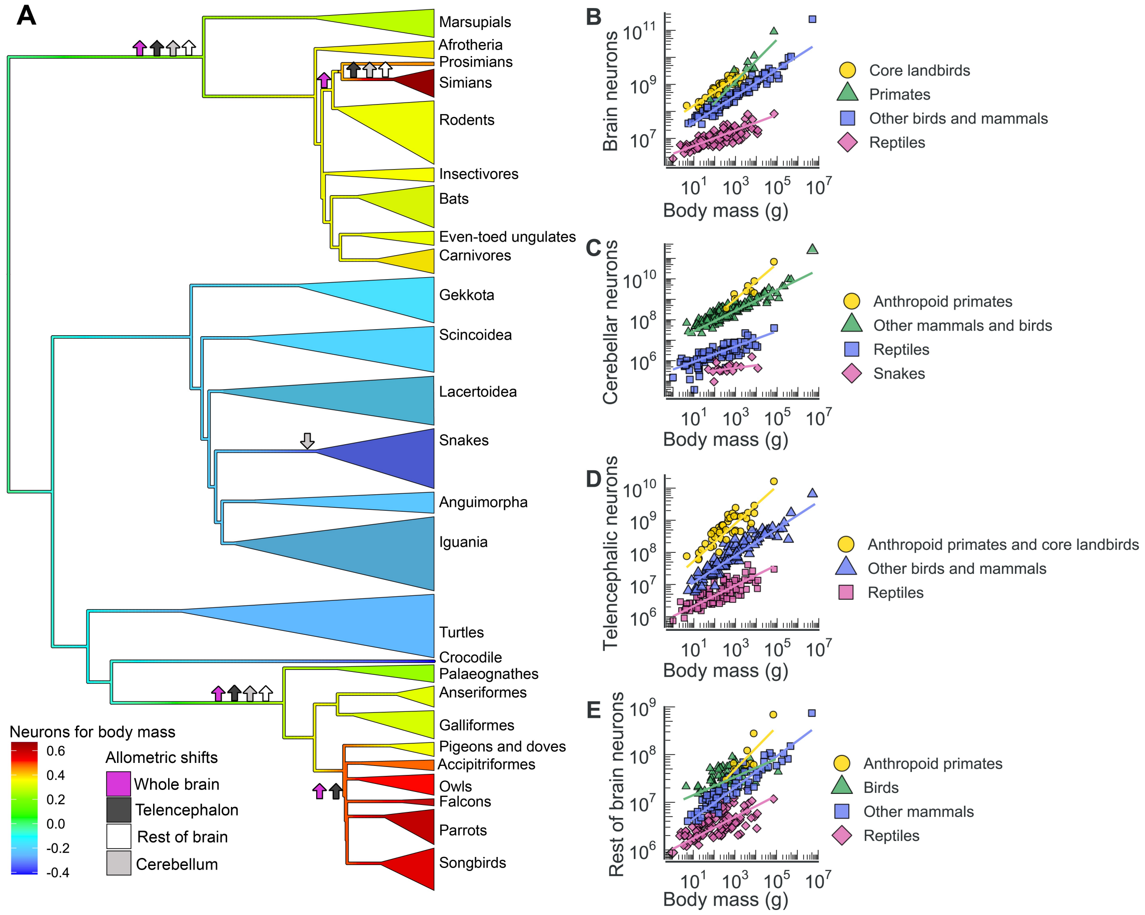

Supplementary Figure 7. Shifts in neurons-body scaling in amniotes. **(A)** Tree colors correspond to neuron numbers relative to body mass, with blue colors indicating low neuron numbers and red colors high neuron numbers. The arrows indicate the branches with shifts in allometric relationship between body mass and neuron number (resulting in either an increase in neurons – arrow up, or a decrease in neurons – arrow down) for the whole brain, telencephalon, cerebellum and rest of brain, identified by reversible-jump Markov chain Monte Carlo analysis with posterior probability > 0.7 for clades including more than 3 species. **(B-E)** Log-log plots of neuron number for body mass with regression lines for the distinct regimes identified by PGLS analysis.

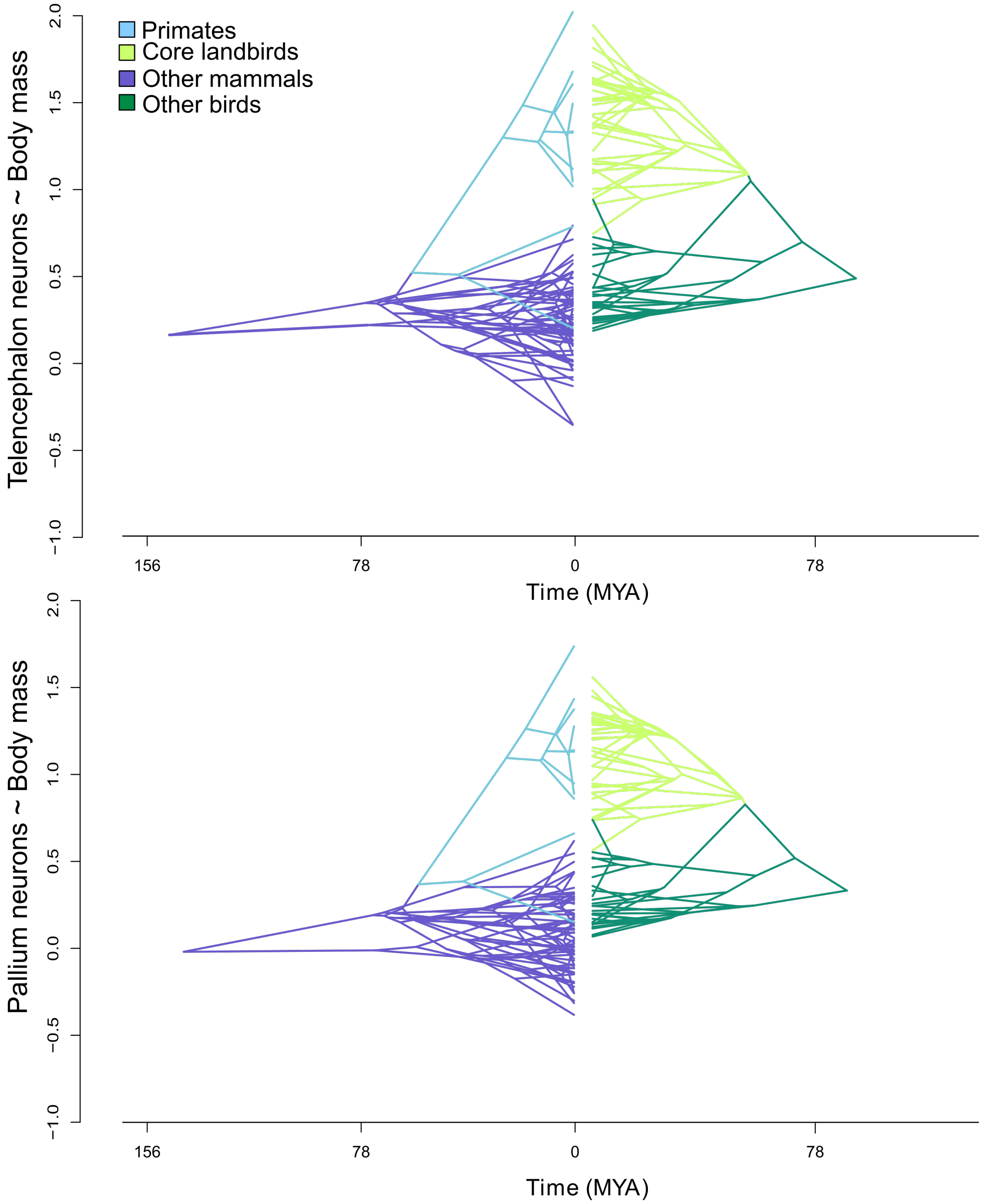

Supplementary Figure 8. Phenograms showing the evolution of telencephalic and pallial neuron numbers relative to body mass over time. Numbers of telencephalic and pallial neurons relative to body mass in mammals and birds are plotted side by side for comparison. The x-axis is flipped in birds, so that 0 (the present) is in the middle and the axis extends symmetrically left and right

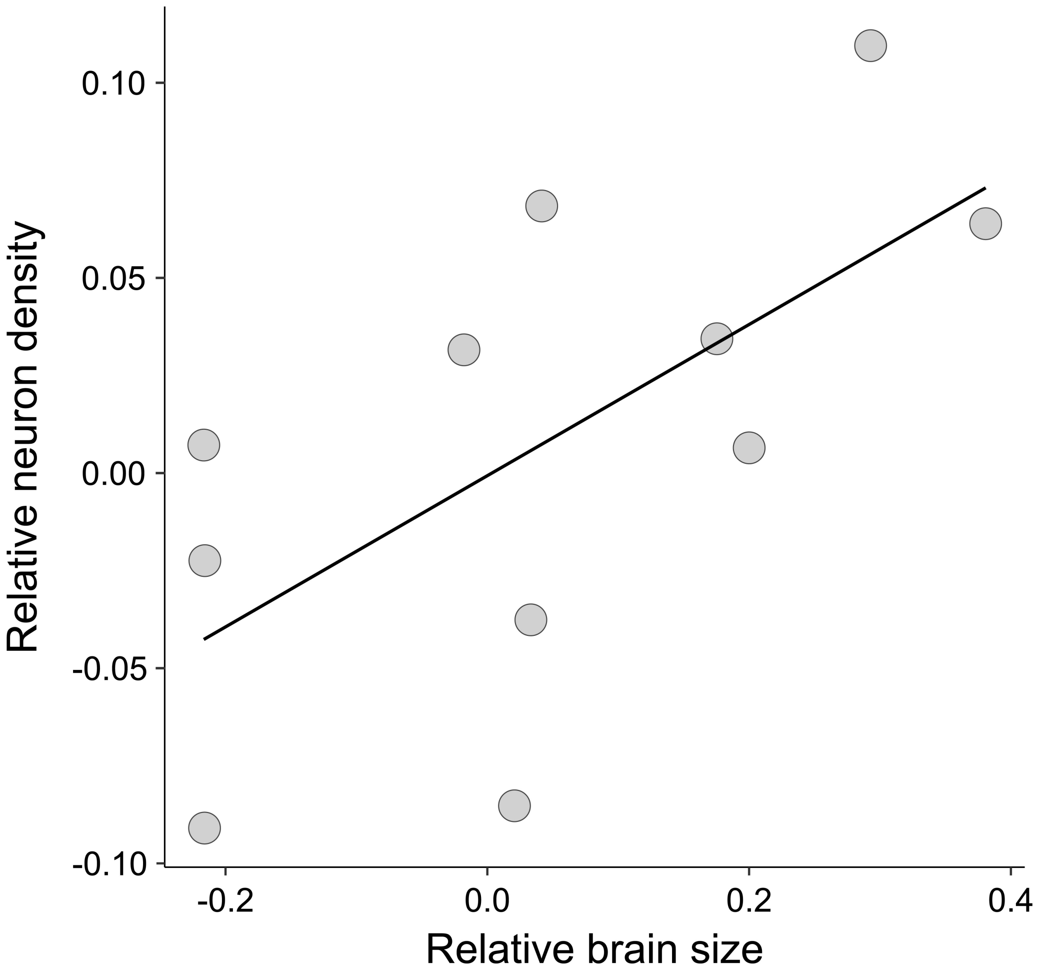

Supplementary Figure 9. Large relative brain size is positively associated with high relative neuron density within primates. Relative brain size and relative neuron density refer to residuals from regression of neuron density on brain size and brain mass on body mass, respectively. The line is a PGLS regression line for primates.

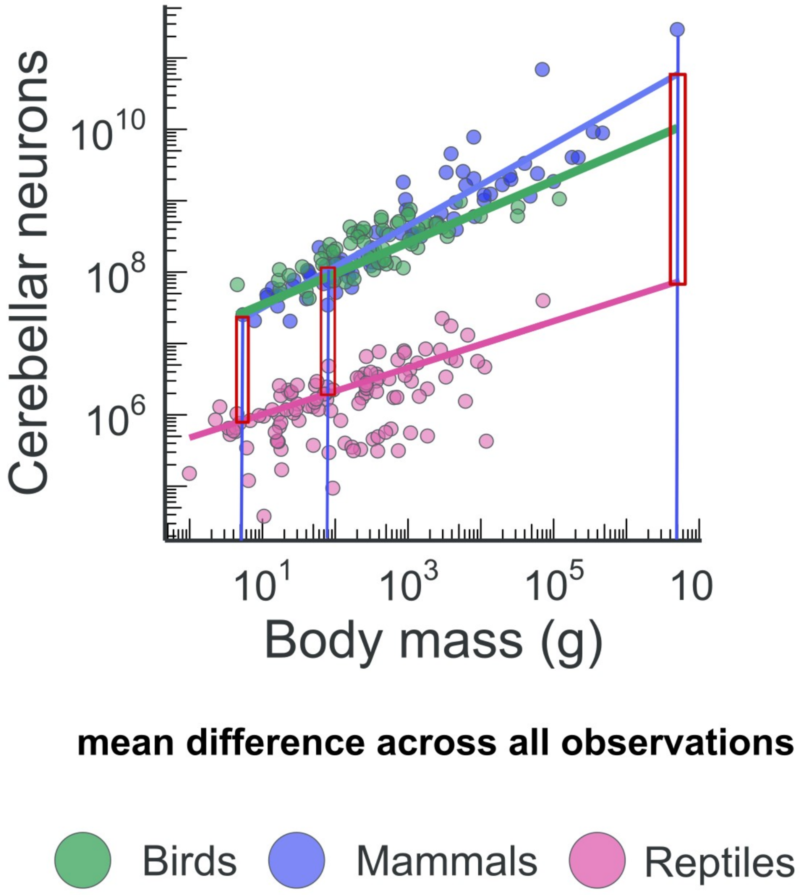

Supplementary Figure 10. Example of how the fold change in the number of neurons for body mass was calculated.

Table S1. Brain mass and neuron number scaling with body mass for birds, mammals and non-avian reptiles.

Group	Intercept±SE	Slope±SE	Mean difference	P-value	Pagel's λ
Brain mass ~ Body mass					
Birds	-1.125±0.121	0.592±0.022	7.2x	<0.001	1.049
Mammals	-1.311±0.191	0.686±0.025	8.42x	<0.001	0.893
Reptiles	-1.688±0.102	0.503±0.018	NA	<0.001	0.849
Brain neurons ~ Body mass					
Birds	7.356±0.15	0.439±0.03	24.99x	<0.001	0.980
Mammals	7.021±0.214	0.545±0.028	22.82x	<0.001	0.900
Reptiles	6.44±0.102	0.288±0.018	NA	<0.001	0.849
Telencephalon neurons ~ Body mass					
Birds	6.874±0.209	0.45±0.041	20.02x	<0.001	0.986
Mammals	6.613±0.249	0.444±0.032	9.48x	<0.001	0.909
Reptiles	6.01±0.113	0.32±0.021	NA	<0.001	0.811
Cerebellum neurons ~ Body mass					
Birds	7.072±0.12	0.44±0.024	51.35x	<0.001	0.974
Mammals	6.797±0.219	0.574±0.029	75.41x	<0.001	0.880
Reptiles	5.666±0.225	0.326±0.036		<0.001	0.923
Rest of brain neurons ~ Body mass					
Birds	6.936±0.107	0.19±0.022	6.91x	<0.001	0.961
Mammals	6.276±0.136	0.348±0.022	4.27x	<0.001	0.703
Reptiles	6.016±0.099	0.225±0.018	NA	<0.001	0.854

The scaling coefficients are from PGLS regression of log10-transformed values. The mean difference refers to the difference in trait mean relative to body mass compared to reptiles. NA, "not applicable".

Table S2. Neuron-brain structure scaling rules for the allometric grades identified by PGLS analysis.

Grade	Intercept±SE	Slope±SE	Pagel's λ	ΔAIC	P-value
Brain neurons ~ Brain mass					
Reptiles	7.41±0.075	0.602±0.027	0.77	74	<0.001
Other birds and mammals	8.096±0.098	0.751±0.031			
Core landbirds and anthropoids	8.281±0.107	0.820±0.039			
Telencephalic neurons ~ Telencephalon mass					
Reptiles	7.191±0.085	0.613±0.031	0.70	85	<0.001
Other birds and mammals	7.734±0.111	0.613±0.035			
Core landbirds and anthropoids	8.088±0.119	0.783±0.045			
Cerebellar neurons ~ Cerebellum mass					
Reptiles	7.767±0.137	0.693±0.029	0.94	28	<0.001
Birds and mammals	8.589±0.172	0.844±0.034			
Rest of brain neurons ~ Rest of brain mass					
Reptiles, marsupials, carnivores and even-toed ungulates	7.076±0.05	0.53±0.022	0.57	46	<0.001
Other birds	7.539±0.091	0.41±0.048			
Placental mammals	7.28±0.048	0.623±0.031			
Songbirds	7.72±0.105	0.511±0.08			

ΔAIC and p-value refer to the comparison between the best-fit model and the null model (no allometric shifts).

Table S3. Neuron-body scaling rules for the allometric grades identified by PGLS analysis.

Grade	Intercept±SE	Slope±SE	Pagel's λ	ΔAIC	P-value
Brain neurons ~ Body mass					
Reptiles	6.446±0.133	0.29±0.022	0.80	138	<0.001
Other birds and mammals	7.085±0.175	0.498±0.028			
Primates	7.705±0.201	0.493±0.044			
Core landbirds	6.626±0.331	0.826±0.078			
Telencephalic neurons ~ Body mass					
Reptiles	5.997±0.158	0.32±0.026	0.81	114	<0.001
Other birds and mammals	6.705±0.207	0.42±0.032			
Core landbirds and monkeys	7.176±0.231	0.571±0.046			
Cerebellum neurons ~ Body mass					
Reptiles	5.591±0.228	0.374±0.034	0.92	81	<0.001
Other birds and mammals	6.913±0.295	0.507±0.04			
Anthropoid primates	6.248±0.482	0.879±0.102			
Snakes	5.365±0.315	0.126±0.086			
Rest of brain neurons ~ Body mass					
Reptiles	6.009±0.121	0.227±0.02	0.78	66	<0.001
Birds	6.995±0.193	0.185±0.032			
Other mammals	6.327±0.206	0.327±0.026			
Anthropoid primates	6.01±0.329	0.537±0.071			

ΔAIC and p-value refer to the comparison between the best-fit model and the null model (no allometric shifts).

Table S4. Neuron-brain structure scaling rules for the allometric grades identified by PGLS analysis in the dataset with imputed data for olfactory bulbs and striatum.

Grade	Intercept±SE	Slope±SE	Pagel's λ	ΔAIC	P-value
Brain neurons ~ Brain mass					
Reptiles	7.41±0.075	0.602±0.027	0.73	78	<0.001
Other birds and mammals	8.1±0.091	0.751±0.03			
Core landbirds and anthropoids	8.287±0.098	0.816±0.038			
Telencephalic neurons ~ Telencephalon mass					
Reptiles	7.191±0.085	0.613±0.031	0.7	87	<0.001
Other birds and mammals	7.743±0.106	0.618±0.035			
Core landbirds and anthropoids	8.097±0.115	0.779±0.044			
Cerebellar neurons ~ Cerebellum mass					
Reptiles	7.767±0.137	0.693±0.029	0.94	28	<0.001
Birds and mammals	8.589±0.172	0.844±0.034			
Rest of brain neurons ~ Rest of brain mass					
Reptiles, marsupials, carnivores and even-toed ungulates	7.045±0.039	0.516±0.022	0.32	41	<0.001
Other birds	7.531±0.071	0.41±0.053			
Placental mammals	7.243±0.043	0.588±0.032			
Songbirds	7.72±0.105	0.511±0.08			

ΔAIC and p-value refer to the comparison between the best-fit model and the null model (no allometric shifts).

Table S5. Neuron-body scaling rules for the allometric grades identified by PGLS analysis in the dataset with imputed data for olfactory bulbs and striatum.

Grade	Intercept±SE	Slope±SE	Pagel's λ	ΔAIC	P-value
Brain neurons ~ Body mass					
Reptiles	6.446±0.133	0.29±0.022	0.78	146	<0.001
Other birds and mammals	7.072±0.167	0.502±0.027			
Primates	7.707±0.192	0.492±0.043			
Core landbirds	6.815±0.257	0.783±0.063			
Telencephalic neurons ~ Body mass					
Reptiles	6.002±0.157	0.32±0.026	0.82	115	<0.001
Other birds and mammals	6.727±0.206	0.42±0.032			
Core landbirds and anthropoids	7.196±0.23	0.569±0.045			
Cerebellum neurons ~ Body mass					
Reptiles	5.591±0.228	0.374±0.034	0.92	81	<0.001
Other birds and mammals	6.913±0.295	0.507±0.04			
Anthropoid primates	6.248±0.482	0.879±0.102			
Snakes	5.365±0.315	0.126±0.086			
Rest of brain neurons ~ Body mass					
Reptiles	6.007±0.119	0.228±0.021	0.7	48.5	<0.001
Birds	7.012±0.191	0.181±0.034			
Other mammals	6.275±0.2	0.312±0.028			
Anthropoid primates	5.73±0.343	0.542±0.078			

ΔAIC and p-value refer to the comparison between the best-fit model and the null model (no allometric shifts).

Table S6. Comparison of rates of neuron-structure mass evolution in the identified grades. σ^2 indicates the rate of evolution of allometric residuals.

Group	σ^2	Reptiles	Other birds	Other mammals	Primates	Core landbirds
Brain neurons ~ Brain mass						
Reptiles	0.0002	NA	0.8	0.38***	0.26**	0.77
Other birds	0.0003	1.24	NA	0.48*	0.32*	0.95
Other mammals	0.0005	2.61***	2.1*	NA	0.67	2*
Primates	0.0008	3.91**	3.15*	1.5	NA	2.99*
Core landbirds	0.0003	1.31	1.05	0.5*	0.33*	NA
Telencephalon neurons ~ Telencephalon mass						
Reptiles	0.0002	NA	0.4**	0.22***	0.12***	0.31***
Other birds	0.0005	2.48**	NA	0.53	0.29*	0.77
Other mammals	0.0010	4.65***	1.87.	NA	0.55	1.45
Primates	0.0018	8.43***	3.4*	1.81	NA	2.63.
Core landbirds	0.0007	3.21***	1.29	0.69	0.38	NA
Cerebellum neurons ~ Cerebellum mass						
Reptiles	0.0005	NA	2.45**	1.77*	0.29*	2.86***
Other birds	0.0002	0.41**	NA	0.72	0.12***	1.17
Other mammals	0.0003	0.56*	1.38	NA	0.16***	1.61
Primates	0.0016	3.49*	8.56***	6.2***	NA	10.01***
Core landbirds	0.0002	0.35***	0.85	0.62	0.1***	NA
Rest of brain neurons ~ Rest of brain mass						
Reptiles	0.0002	NA	0.46*	0.19***	0.08***	0.73
Other birds	0.0004	2.2*	NA	0.41**	0.17**	1.61
Other mammals	0.0009	5.33***	2.43**	NA	0.41.	3.92***
Primates	0.0021	13.06***	5.94**	2.45	NA	9.59***
Core landbirds	0.0002	1.36	0.62	0.26***	0.1***	NA

Values represent ratios between rates of the group in the row and the groups in the columns. NA means "not applicable". Statistically significant differences between groups: ***, p < 0.001; **, p 0.01 – 0.001; *, p 0.01 – 0.05; no symbol, p > 0.05

Table S7. Comparison of rates of neuron-body evolution in the identified grades. σ^2 indicates the rate of evolution of allometric residuals.

Group	σ^2	Reptiles	Other birds	Other mammals	Primates	Core landbirds
Brain neurons ~ Body mass						
Reptiles	0.0005	NA	1.25	0.61*	0.07***	0.67
Birds	0.0004	0.8	NA	0.49*	0.06***	0.54.
Other mammals	0.0009	1.65*	2.06*	NA	0.12***	1.11
Primates	0.0077	14.13***	17.68***	8.59***	NA	9.52***
Core landbirds	0.0008	1.48	1.86.	0.9	0.11***	NA
Telencephalon neurons ~ Body mass						
Reptiles	0.0005	NA	0.59	0.37***	0.06***	0.31***
Birds	0.0008	1.69.	NA	0.62	0.1***	0.53.
Other mammals	0.0013	2.73***	1.62	NA	0.17**	0.86
Primates	0.0079	16.54***	9.79***	6.05**	NA	5.18**
Core landbirds	0.0015	3.19***	1.89.	1.17	0.19**	NA
Cerebellum neurons ~ Body mass						
Reptiles	0.0013	NA	3.17***	1.27	0.14***	2.37**
Birds	0.0004	0.32***	NA	0.4**	0.04***	0.75
Other mammals	0.0010	0.79	2.49**	NA	0.11***	1.86*
Primates	0.0093	7.11***	22.54***	9.05***	NA	16.82***
Core landbirds	0.0006	0.42**	1.34	0.54*	0.06***	NA
Resto of brain neurons ~ Body mass						
Reptiles	0.0003	NA	0.6.	0.28***	0.05***	0.82
Birds	0.0005	1.67.	NA	0.47*	0.08***	1.36
Other mammals	0.0011	3.55***	2.13*	NA	0.18**	2.91***
Primates	0.0062	19.94***	11.96***	5.61**	NA	16.32***
Core landbirds	0.0004	1.22	0.73	0.34***	0.06***	NA

Values represent ratios between rates of the group in the row and the groups in the columns. NA means "not applicable". Statistically significant differences between groups: ***, p < 0.001; **, p 0.01 – 0.001; *, p 0.01 – 0.05; no symbol, p > 0.05.

Table S8. Comparison of rates of neuron-structure mass evolution of different brain structures in non-avian reptiles, birds and mammals.

Structure	σ^2	Telencephalon	Cerebellum	Rest of brain
Reptiles				
Telencephalon	0.0011	NA	0.35***	1.45
Cerebellum	0.0003	2.86***	NA	4.15***
Rest of brain	0.0004	0.69	0.24***	NA
Birds				
Telencephalon	0.0004	NA	2.93***	3.56***
Cerebellum	0.0003	0.34***	NA	1.21
Rest of brain	0.0012	0.28***	0.82	NA
Mammals				
Telencephalon	0.0021	NA	1.1	1.32
Cerebellum	0.0017	0.91	NA	1.2
Rest of brain	0.0023	0.75	0.83	NA

σ^2 indicates the rate of evolution of allometric residuals. Values represent ratios between rates for the structure in the row and the structures in the columns. NA means "not applicable". Statistically significant differences between groups: ***, p < 0.001; **, p 0.01 – 0.001; *, p 0.01 – 0.05; no symbol, p > 0.05.

Dataset S1 (separate file). Numbers of neurons and non-neuronal cells in the investigated brain parts for 145 species of birds and non-avian reptiles.

Dataset S2 (separate file). Brain and body masses for 149 species of non-avian reptiles.
